# HARTH: A Human Activity Recognition Dataset for Machine Learning

**DOI:** 10.3390/s21237853

**Published:** 2021-11-25

**Authors:** Aleksej Logacjov, Kerstin Bach, Atle Kongsvold, Hilde Bremseth Bårdstu, Paul Jarle Mork

**Affiliations:** 1 Department of Computer Science, Faculty of Information Technology and Electrical Engineering, Norwegian University of Science and Technology, 7034 Trondheim, Norway; kerstin.bach@ntnu.no; 2 Department of Public Health and Nursing, Faculty of Medicine and Health Sciences, Norwegian University of Science and Technology, 7034 Trondheim, Norway; atle.m.kongsvold@ntnu.no (A.K.); paul.mork@ntnu.no (P.J.M.); 3 Department of Neuromedicine and Movement Science, Faculty of Medicine and Health Sciences, Norwegian University of Science and Technology, 7034 Trondheim, Norway; hilde.bardstu@ntnu.no; 4 Department of Sport, Food and Natural Sciences, Faculty of Education, Arts and Sports, Western Norway University of Applied Sciences, 6851 Sogndal, Norway

**Keywords:** physical activity behavior, human activity recognition, public dataset, benchmark, machine learning, deep learning, accelerometer

## Abstract

Existing accelerometer-based human activity recognition (HAR) benchmark datasets that were recorded during free living suffer from non-fixed sensor placement, the usage of only one sensor, and unreliable annotations. We make two contributions in this work. First, we present the publicly available Human Activity Recognition Trondheim dataset (HARTH). Twenty-two participants were recorded for 90 to 120 min during their regular working hours using two three-axial accelerometers, attached to the thigh and lower back, and a chest-mounted camera. Experts annotated the data independently using the camera’s video signal and achieved high inter-rater agreement (Fleiss’ Kappa =0.96). They labeled twelve activities. The second contribution of this paper is the training of seven different baseline machine learning models for HAR on our dataset. We used a support vector machine, k-nearest neighbor, random forest, extreme gradient boost, convolutional neural network, bidirectional long short-term memory, and convolutional neural network with multi-resolution blocks. The support vector machine achieved the best results with an F1-score of 0.81 (standard deviation: ±0.18), recall of 0.85±0.13, and precision of 0.79±0.22 in a leave-one-subject-out cross-validation. Our highly professional recordings and annotations provide a promising benchmark dataset for researchers to develop innovative machine learning approaches for precise HAR in free living.

## 1. Introduction

Physical activity behavior has a major influence on public health [1,2]. However, studies investigating the effect of physical behavior on disease risk often rely on self-reported data, which are susceptible to bias and misclassification [3,4]. Objective measurements can overcome some of the shortcomings of self-reported data [5,6]. Human activity recognition (HAR) is a field of study that focuses on recognizing specific human physical activities and postures based on sensor data [7,8]. Body-worn accelerometers are the most commonly used data collection method to support HAR due to their low cost and small size [9]. Several studies have shown that body-worn accelerometers provide valid information of human physical activity and postures [10]. Over the last ten years, machine learning approaches have become common to classify sensor data for HAR [11,12,13].

Different studies have trained and tested their machine learning models on self-recorded datasets, but only a few of these sets are publicly available [9,14,15,16]. However, an objective comparison between different machine learning approaches is only possible if such datasets become publicly available [17]. Additionally, most machine learning studies for accelerometer-based HAR were performed in a laboratory setting or controlled environment [18]. Several studies have shown that machine learning models developed in laboratory conditions demonstrate poor performance when tested outside the laboratory [13,19,20,21]. Only a few studies have been conducted in free-living conditions, i.e., where participants are free to perform activities of their everyday life but have to perform certain predefined activities at least once. Even fewer of these studies have investigated data from two accelerometers [12,13,18], although several studies showed that the utilization of more than one sensor could considerably improve the classification performance [10,13,22,23]. The major drawback of existing free-living datasets is the trustworthiness of the activity annotations, as the related publications do not report the reliability of the annotation method [24,25,26,27,28,29,30]. Poor training data annotations hamper the possibility of training machine learning models.

Free-living activity data from approximately 35,000 people have been recorded in the fourth round of Norway’s biggest health study, the Trøndelag Health Study (HUNT4) [31,32]. The data was collected over seven days using two body-worn three-axis accelerometers located on the participants’ thigh and lower back [31]. Performing HAR on this dataset facilitates research that will bring new insights into the association between physical activity behavior and public health. Hence, it is essential that HAR models are trained on annotated datasets that resemble the HUNT4 accelerometer data.

Two contributions are made in this paper. First, we present the Human Activity Recognition Trondheim dataset (HARTH). Twenty-two participants performed different activities during their regular working hours while carrying out their everyday activities as naturally as possible. Two experts annotated twelve activities in total. We used two accelerometers placed on the thigh and lower back to collect sensor data. HARTH provides high-quality acceleration measurements with fixed sensor placements and professionally annotated labels. To the best of our knowledge, the combination of these three factors is not yet considered by other accelerometer-based and publicly available free-living HAR datasets. HARTH is publicly available to enable an objective comparison between HAR models for future research (https://github.com/ntnu-ai-lab/harth-ml-experiments, accessed on 16 November 2021). Second, we train seven different baseline classification models on HARTH, including (1) the k-nearest neighbors (k-NN), (2) the support vector machine (SVM), (3) the random forest (RF), (4) the extreme gradient boost (XGB), (5) the bidirectional long short-term memory (BiLSTM), (6) the convolutional neural network (CNN), and (7) a CNN with multi-resolution modules.

With this work, we want to encourage researchers to use the presented machine learning models (or potential future models) to perform health studies based on physical activity behavior. Previous works [12,13] and international projects [33,34,35] already showed great interest in such studies, as they share the same recording setup used in this work.

This paper is organized as follows. Section 2 gives an overview of publicly available free-living datasets as well as of related HAR works. The HARTH and the utilized baseline machine learning models are presented in Section 3. The experimental setup and results are presented in Section 4. We discuss our results in Section 5 and provide conclusions and future work in Section 6.

## 2. Related Work

### 2.1. Public Har Datasets

According to Micucci et al. [14], and Reiss and Stricker [15] few accelerometer-based datasets for HAR are publicly available. This was also confirmed in a recent survey [9], showing that only 30 of 142 accelerometer-based datasets were publicly available. However, few of these datasets can be considered to be recorded during free-living. We found 62 accelerometer-based HAR datasets, but only eight of them can be considered free-living. We summarize these datasets in Table 1.

Garcia-Gonzalez et al. [24] proposed an orientation-, placement-, and subject-indepen dent dataset, called Real-life-HAR, where 19 participants performed four activities while carrying a smartphone (Real-life-HAR available at: https://lbd.udc.es/research/real-life-HAR-dataset (accessed on 22 April 2021)). They did not specify the smartphone placement. The participants were free to perform the activities whenever they wanted during their everyday life. They were only asked to annotate the beginning and the end of the activity using an app on their smartphone. The activities were: inactive (not carrying the phone), active, walking/running, and driving. “Active” means that the person carried the phone but did not walk (e.g., standing while doing laundry). Driving includes all types of engine-based transportation. The dataset considers two physical activities, namely walking and standing/sitting (included in driving and active).

In the Sussex-Huawei Locomotion (SHL) dataset [36,37], three subjects carried four smartphones and a camera (chest-mounted) while performing eight different transportation activities, namely: being still (no transportation), walking, running, cycling, driving a car, taking the bus, taking the train, and being in a subway (SHL dataset available at: http://www.shl-dataset.org (accessed on 22 April 2021)). Annotations were created during the data collection using one smartphone. The labels were validated after the data collection using the camera’s video signal. The data were recorded over several days, and instructions were given on what to perform each day. However, the subjects were able to decide when and where to perform the activities. Furthermore, they were free to perform activities of their everyday life. Four physical activities are considered in the dataset: walking, running, cycling, and sitting/standing. The transportation activity “still” includes both standing and sitting, making it impossible to distinguish them.

The HASC-PAC2016 [25] is a collection of previously published HASC-PAC datasets [39,40,41,42] (The HASC-PAC2016 is available at: http://hub.hasc.jp/corpora (accessed on 22 April 2021)). Eighty-one subjects were recorded in an everyday life setting. They were free to perform six activities whenever they wanted in their daily lives as long as they were performed between landmarks, i.e., user-defined start and end geographical locations. The activities were no activity (standing/sitting/lying), walking, running, skipping, and walking stairs. A smartphone accelerometer was used for data acquisition, and annotations were performed via an app. It was not specified where to wear the smartphone or which manufacturer to use.

A smartphone and an app were also used to record accelerometer data for the WISDMv2.0 dataset [26,27] (WISDMv2.0 available at: https://www.cis.fordham.edu/wisdm/dataset.php#actitracker (accessed on 22 April 2021)). Users recorded data during everyday life while carrying the smartphone. They were free to annotate particular activities by themselves [17] or leave specific movements unlabeled. The annotated activities include walking, running, stair climbing, sitting, standing, and lying. At the time of writing this work, 323 users provided acceleration data, while 225 of them annotated parts of their daily activities. The activity types standing, lying, and sitting are distinguished.

Several parameters were recorded and annotated in the DailyLog dataset [28], including the environmental context, the sensor position, and nineteen activities with 33 sub-activities (DailyLog available at https://sensor.informatik.uni-mannheim.de/#dataset_dailylog (accessed on 23 April 2021)). The seven considered physical activities are: climbing, jumping, lying, running, sitting, standing, and walking. Higher-level activities like sports were examined as well but not considered different physical activities as they combine multiple basic activities. A smartphone and a smartwatch were used for recordings. Seven participants recorded their daily routine (≈10 h) for several days and annotated the data via an app on the smartphone.

In the ExtraSensory [29] dataset, 60 participants used an app on their smartphones to annotate different labels during approximately one week of their everyday lives (ExtraSensory available at http://extrasensory.ucsd.edu (accessed on 12 May 2021)). Acceleration data were recorded using the smartphone and a smartwatch. Recordings were performed in 20-s windows every minute. Hence there are gaps between measurements. The dataset includes 51 different labels, with eight of them being physical activities, namely sitting, lying, standing, walking, cycling, running, and walking upstairs/downstairs.

The TMD dataset of Carpineti et al. [30] is primarily created for transportation mode detection tasks with four different types of transportation (bus, car, train, and walking) and standing still (TMD available at http://cs.unibo.it/projects/us-tm2017 (accessed on 12 May 2021)). However, as the dataset comprises the three physical activities walking, standing still, and sitting (in a car), we consider it here. Smartphone sensors were used to record multiple modalities, including acceleration. The 13 participants used a smartphone app to label the data during their daily activities.

Herrera-Alcántara et al. [38] created a dataset containing ten different daily-living activities of eight students. We refer to this dataset as Students’ Daily Living (short: SDL) (Students’ DailyLiving available upon request to the corresponding authors). Acceleration data were recorded using a smartwatch, and annotations were performed by the students using a smartphone app. The activities are eating, running, sleeping, classroom-session, exam, job, homework, transportation, watching TV (series), and reading. We can identify four possible physical activities, namely, sitting, standing, running, and walking. Currently, this dataset is only available upon request to the corresponding authors.

The presented datasets have several limitations. First, most of them were recorded using smartphones. Smartphone accelerometers generally suffer from low sensitivity and a high output noise level [43]. Second, their exact positions were not always fixed [24,25,28]. Without a fixed sensor placement, the same activity can look considerably different in the signal, which can lead to high intra-class variance and poor HAR performance [44]. Third, except for the SHL, none of the publications related to the available datasets report the reliability of the annotation method. This is because the users annotated the labels. Poor quality of the training data may hamper the possibility to train machine learning models for HAR.

### 2.2. Human Activity Recognition Approaches

Few HAR research papers investigate more than one accelerometer, even though classification performance can be improved if doing so [10,13,22,23]. We present related machine learning-based HAR works that examine more than one accelerometer but do not use additional sensors (e.g., gyroscopes). We further focus only on activities similar to ours.

Stewart et al. [12] trained an RF classifier using an in-lab recorded dataset of 75 (42 children, 33 adults) participants wearing two Axivity AX3 (Axivity Ltd., Newcastle, UK) [45] accelerometers on the thigh and lower back. The six activities, sitting, lying, standing, slow walking, fast walking, and running, were predicted with a balanced accuracy of 99.1% for adults and 97.3% for children. A similar study was made by Narayanan et al. [13]. Free-living data of 30 participants (15 children, 15 adults) that wore the same AX3 accelerometers on the thigh, lower back, and wrist, were recorded. After different sensor position combinations were compared, the thigh/lower back combination led to the best balanced accuracy of 95.6% (adults) and 92% (children) using an RF classifier. Bao and Intille [46] investigated up to five bi-axial accelerometers (right hip, dominant wrist, non-dominant upper arm, dominant ankle, non-dominant thigh) worn by 20 subjects who performed 20 activities. Four classifiers were compared, while the decision tree showed the best results (84%). Bao and Intille [46] concluded that even though five accelerometers led to the best results, two sensors are sufficient for certain activities. A similar conclusion was made by Olguín and Pentland [23]. They used acceleration data of up to three sensors (wrist, hip, chest). Using all three led to the best accuracy (92.1%), but using only two can show similar results of 87.2% (wrist, hip). Hip/wrist configurations were also examined in [20]. The authors trained an RF classifier on free-living data of preschool-aged childrens’ activities.The combination of hip and wrist accelerometers showed a better F-score than the two sensors individually. Shoaib et al. [47] used a smartphone and smartwatch for data acquisition and an SVM, a k-NN, and a decision tree to recognize seven activities. The combination of both sensors outperformed the individual ones for certain activities. By training four classifiers (k-NN, SVM, decision tree, naïve Bayes), Gao et al. [48] showed that a combination of thigh-, chest-, side-, and waist-mounted accelerometers performed better than each sensor individually. Shoaib et al. [49] investigated seven machine learning models (naïve Bayes, decision tree, RF, Bayesian network, SVM, logistic regression, k-NN) to classify seven activities. For data acquisition, five smartphones (right/left trouser pocket, belt, right upper arm, right wrist) were used. Nine accelerometers (left/right ankle, left/right hip, left/right upper arm, left/right wrist, spine) were used in the work of Fullerton et al. [10]. A k-NN, a decision tree, an SVM, and an ensemble-bagged tree method were trained to predict six activities. The former model achieved the best results with 97.6% accuracy. Baños et al. [50] also investigated nine sensors (each body limb and upper back) and trained a k-NN (best), a decision tree, and a nearest class center classifier. Maurer et al. [51] trained a k-NN, a decision tree, a naïve Bayes, and a Bayesian network on a dataset recorded with six bi-axial accelerometers. Six subjects performed six activities. Each sensor position is analyzed separately. The best acceleration-based results were 76.6% (wrist), 79.5% (pocket), 87.2% (bag), 72.6%, (necklace), 78.0% (shirt), and 77.2% (belt) using the decision tree. An AdaBoost classifier and four accelerometers were used by Ugulino et al. [52] to classify five activities. The best overall weighted accuracy was 99.4%. Zubair et al. [53] used the same dataset as Ugulino et al. [52] to train an RF and AdaBoost classifier. The former outperformed the latter with an overall accuracy of 99.9%, an averaged precision, and recall of 99.8, respectively. More recently, Gupta et al. [54] proposed a combination of time CNN and stacked LSTM model and compared it with three other deep learning models on a dataset containing nine activities. It was recorded using three accelerometers placed on the backs of seven subjects. The proposed model outperformed the others with an average accuracy of 99.77%. Further studies investigating multiple accelerometers are [55,56] (six sensors), [57,58] (four sensors, dataset of Ugulino et al. [52]), [59] (nine sensors, dataset of Baños et al. [50]), and [60] (two sensors).

Most presented works used more than two sensors, but as Bao and Intille [46] and Olguín and Pentland [23] mentioned, doing so does not improve the HAR results considerably. Furthermore, using a lower number of sensors also creates a better level of comfort for participants.

## 3. Methods

### 3.1. Human Activity Recognition Trondheim Dataset

The main characteristics of HARTH are summarized in Table 1. We used two tri-axial Axivity AX3 accelerometers (Axivity Ltd., Newcastle, UK) [45] for data acquisition. The AX3 is a small (23×32.5×7.6 mm) and lightweight (11 g) sensor. The sampling rate (12.5–3200 Hz), the measurement range (±2/4/8/16 g), and the resolution (up to 13 bit) are configurable. Accelerometer data are stored locally on a 512 Mb flash memory chip and can be transferred via a Micro-B USB connector. Additionally, each AX3 is equipped with a temperature and ambient light sensor. There are several reasons why we use two sensors. First, Cleland et al. [22] investigated up to six sensors but observed no significant increase in performance compared to two sensors. The same findings are observable in the work of Awais et al. [61]. Hence, two sensors provide high accuracy, higher comfort for the participants [15], and reduced costs [13]. Second, previous works showed promising results using two AX3 with similar sensor positions [12,13]. Third, a classifier trained on HARTH can lead to promising predictions on the previously mentioned HUNT4 dataset. The term “HARTH” is the abbreviation for “Human Activity Recognition Trondheim.” It is named after the place it was recorded.

**Figure 1:**
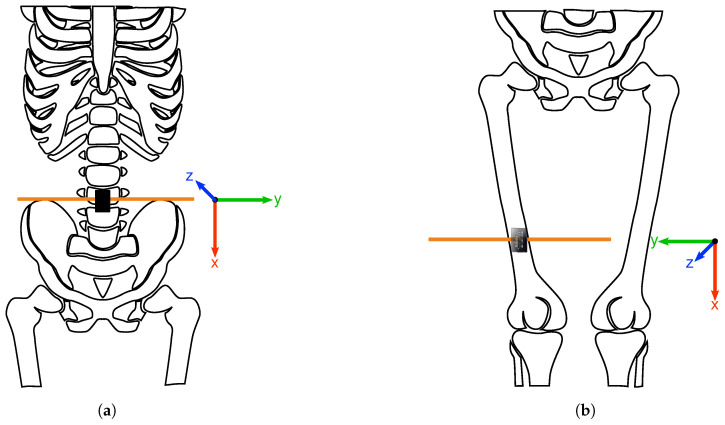
This figure shows the two sensor positions (highlighted with orange lines) used for our dataset. (**a**) The lower back sensor is positioned at approximately the 3rd lumbar vertebra. The z-axis of the coordinate system points forward. (**b**) The thigh sensor is positioned approximately 10 cm above the upper kneecap. The z-axis points backward.

As illustrated in Figure 1, one sensor was attached to each participant’s right, front thigh (≈10 cm above the upper kneecap), and the other to their lower back (approximately 3rd lumbar vertebra). The AX3 are aligned vertically, with the USB connector pointing downward and the side without writing mounted against the skin. Hence, seen from the participant’s perspective while standing upright, the lower back sensor’s x-axis points downward, the y-axis to the left, and the z-axis forward. For the thigh sensor, the y-axis points to the right and the z-axis backward.

A video camera (GoPro Hero3+ [62]) was placed on each participant’s chest using a chest harness, pointing downwards to record leg movements, later used for annotation. We recorded with a frame rate of 30 fps and a resolution of 1280×720 pixels. Twenty-two healthy adults (eight female) were recruited via word of mouth between university and hospital staff. They were on average 38.6±14 years old (range: 25–68), had an average height of 177.3±8.3 (range: 157–191) cm, an average weight of 72.9±10.6 (range: 56.0–92.0) kg, and an average BMI of 23.1±2.3 (range: 19.2–28.4) $\mathrm{kg}/m^{2}$. Each participant gave written informed consent, and we obtained ethical approval from the Regional Committee for Ethics in Medical Research (Mid-Norway [2015/1432]).

**Figure 2:**
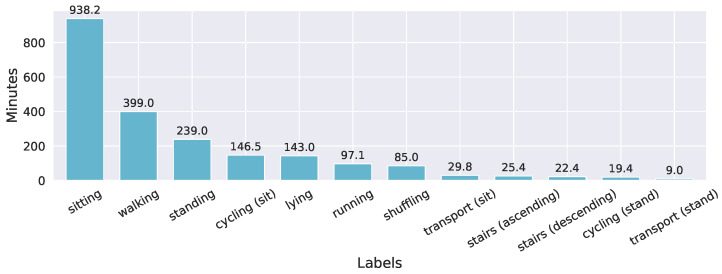
This bar plot shows the total amount of recorded minutes for each activity in the dataset.

We recorded the dataset in two sessions. In the first session, 15 (six female) participants were told to perform their everyday life as normally as possible, during a recording period of 1.5–2 h. They were instructed to perform the activities sitting, standing, lying, walking, and running (including jogging) for at least two to three minutes. During this time, the two sensors recorded acceleration data with a sampling rate of 100 Hz (which we later downsampled to 50 Hz) and a measurement range of ±8 g. At the beginning of the recordings, each participant performed three heel drops (i.e., dropping the heels firmly on the floor), which helped synchronize the acceleration and video signals later. In total, approximately 1804 min (≈30 h) were recorded in the first session. The average recording duration was around 120±21.6 min. When the recordings were finished, the videos were converted to 25 fps and 640×360 pixels and annotated frame-by-frame. Besides the introduced five activities, participants carried out further activities, which we labeled as follows: stairs (ascending), stairs (descending), shuffling (standing with leg movement), cycling (standing), cycling (sitting), transport (sitting) (e.g., in a car), and transport (standing) (e.g., in a bus). This resulted in twelve different labels in total. The labeling was done following a coding scheme with definitions for the different activities, shown in Table A1. After the first session, we observed high imbalances in the class labels; i.e., the distribution was skewed towards light activities. A second data collection session in a free-living setting was therefore carried out with the aim of mainly collecting data on walking, running, and cycling (sitting and standing). All activities included flat, uphill, and downhill sections. There were no further instructions on where and when to carry out the activities. Thus, participants also performed other activities (i.e., sitting, lying, walking stairs), which also were annotated. The second session includes around 417.6 min (≈7 h) of recorded data with an average duration of approximately 60±9 min per participant. The accelerometers’ sampling rate was set to 50 Hz and the measurement range to ±8 g. Human experts annotated the data independently using the ANVIL annotation tool [63]. As a result, they achieved a Fleiss’ Kappa of 0.96. Each file was annotated by at least one expert using the raw data and another person verifying the annotations. Figure 2 summarizes the time distribution of the dataset’s activities in minutes. Although we added the second session, the dataset still shows an imbalance in the labels, making it more challenging to train reliable machine learning models.

**Figure 3:**
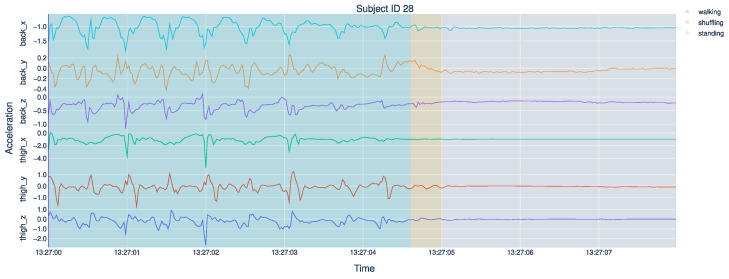
This figure shows ten seconds (x-axis) of the acceleration signals (on the y-axis and in $m/s^{2}$) of all three axes of the back and thigh accelerometers. We focus on the subject with subject ID 28. The background is shaded according to the activity label, in this case walking (green), shuffling (yellow), and standing (gray).

Figure 3 illustrates ten seconds of back and thigh acceleration of one particular subject. The shaded areas represent the different activities of walking, shuffling, and standing in green, yellow, and gray. It is observable that walking exhibits a repeating pattern in all six axes, corresponding to the participant’s steps. After that, the acceleration stabilizes but is not constant. In particular, the thigh_y and thigh_z axes show small repeating patterns, which are expected during shuffling. Standing shows a nearly constant acceleration in all six axes.

### 3.2. Human Activity Recognition Models

We consider seven supervised machine learning approaches for HAR, namely k-NN, SVM, RF, CNN, bidirectional LSTM, extreme gradient boost (XGB), and CNN with multi-resolution modules (multi-resolution CNN). This allows us to provide a good benchmark on how different types of machine learning models perform on HARTH. We cover often-used (former four) and seldom-used (latter three) machine learning approaches for HAR. It also enables the comparison of deep learning and traditional machine learning approaches.

#### 3.2.1. K-Nearest Neighbors

Given a previously observed training set *X* and an unlabeled data sample *x*, the k-nearest neighbors (k-NN) [64] algorithm classifies *x* by computing the (Euclidean) distance to all $x_{tr}\in X$ and choosing the majority label of the k closest training samples. A distance-based weighting can also be applied to increase the closer points’ influence on the final label prediction [65].

#### 3.2.2. Support Vector Machine

The support vector machine (SVM) [66] algorithm creates one or more hyperplanes (decision boundaries) in the n-dimensional input feature space while ensuring that the distance to the nearest samples of each label is maximal. This requires the data to be linearly separable. If the data are not linearly separable, one can project the training data into a higher, N-dimensional space (N > n) and find an optimal hyperplane there. However, such a projection can be computationally expensive. The SVM algorithm uses the *kernel trick* to avoid this problem. Instead of projecting the data points directly into a higher-dimensional space, a kernel function is used that describes the dot-product of data points in that N-dimensional space, which is enough to find an optimal decision boundary.

#### 3.2.3. Random Forest

The random forest (RF) [67,68] algorithm is an ensemble learning technique. Hence, multiple “weak” machine learning models (in this case, decision trees) predict the labels of new input data. The majority label of the weak classifiers’ predictions is then the final prediction of the RF. In addition, random feature selection/subsampling is performed during training. Therefore, each decision tree is only trained on a subset of input features to decrease the correlation between decision trees and increase the generalization capabilities. Furthermore, each weak classifier can be trained on only one subset of randomly selected samples to improve the performance further [68]. This technique is called bootstrapping.

#### 3.2.4. Extreme Gradient Boost

Although the extreme gradient boost (XGB) is seldom used in HAR, it achieves state-of-the-art performance in many other research fields [69]. XGB is a particular implementation of the gradient boosting algorithm [70], an ensemble learning algorithm similar to RF. However, instead of training each weak classifier independently, a sequential learning strategy is utilized. Each weak classifier (in this case, decision trees) tries to correct the previous weak classifier’s errors by minimizing a predefined loss function L using the gradient of L with respect to the previous weak classifier’s prediction [70]. The final prediction of the XGB is the sum of each weak classifier’s prediction, weighted by a learning rate. The XGB adds additional features to the standard gradient boosting, e.g., L1 and L2 regularization.

#### 3.2.5. Bidirectional Long Short-Term Memory

The bidirectional long short-term memory (BiLSTM) [71,72] is an extended version of the standard LSTM [73,74]. An LSTM is a recurrent neural network. It uses both the current input and past activations for training. This allows learning temporal features in a time series across several time frames. Three different gates (input, output, and forget gates) are used in each network cell [74] to mitigate the exploding and vanishing gradient problem that standard recurrent neural networks often suffer from [73]. The different gates control the activation flow through the units and determine how much information should be memorized or forgotten. The BiLSTM uses past, present, and future information for every point in the input time series, exhibiting a larger context, which can be helpful for accelerometer-based HAR. This is achieved by presenting the input stream in forward and backward directions to two separate recurrent hidden layers. Yu and Qin [75] and Nafea et al. [76] investigated them in their works and achieved good HAR results.

#### 3.2.6. Convolutional Neural Network

A major difference between convolutional neural networks (CNNs) and standard neural networks such as like multilayer perceptrons is the weight sharing between successive layers [77]. Instead of connecting each unit of the previous layer with each of the next, a “small” frame of weights (filter) is moved across the input signal (or hidden feature map) while performing a convolution/cross-correlation operation. Usually, a non-linear activation function such as the Rectified Linear Unit (ReLU) is applied afterward [77]. The resulting feature map represents the next layer on which additional filters can be applied. Stacking multiple convolutional layers allows the extraction of low-level features in the first layers and high-level features in layers close to the network’s output [78]. More than one filter is often utilized in one layer to learn to extract multiple features, and the parameters in these filters are trained during backpropagation [77]. Fully connected layers are usually used at the end of the CNN, with the last being the prediction layer with an activation function (e.g., softmax for classification). In contrast to recurrent neural networks, CNNs assume no correlation between input windows and are, therefore, unable to learn long-term dependencies [79]. Instead, they capture local and small changes in the signal using the filters.

#### 3.2.7. Multi-Resolution CNN

**Figure 4:**
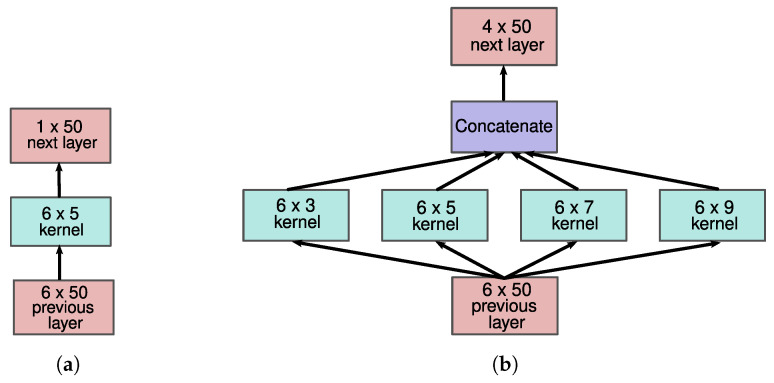
This figure illustrates a single layer in a standard CNN (**a**) and a multi-resolution CNN (**b**).

Nafea et al. [76] showed promising HAR results using multi-resolution modules, which are based on inception modules proposed by Szegedy et al. [80]. This inspired us to investigate them as well. Instead of utilizing one kernel size in a single layer, like the standard CNN (see Figure 4a), multiple kernel sizes are used (see Figure 4b) and concatenated afterward. Hence, features of different scales are extracted in a single layer. Figure 4 illustrates this. Assuming a stride of 1 in our example, each kernel (of both models) produces an 1×50 output. Concatenating them results in a 4×50 output of the multi-resolution module.

### 3.3. Preprocessing

**Figure 5:**
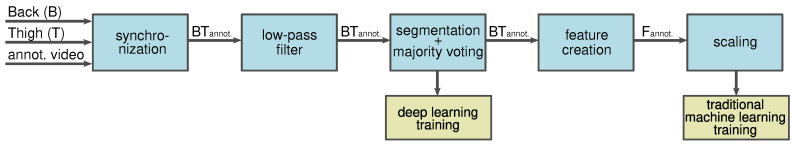
This figure illustrates the five preprocessing steps we performed. First, the two accelerometer signals and the annotated (denoted as annot.) video are time-synchronized. Second, a 20 Hz low-pass filter is applied to the annotated acceleration signals. Third, each signal is segmented into one-second windows, and a majority label voting is used. These windows are fed into the deep learning models for training. Fourth, 161 features (denoted as F) are computed for each window. Fifth, min–max feature scaling is applied. The resulting feature vectors are used to train the traditional machine learning models.

We performed five preprocessing steps before training the machine learning models, as illustrated in Figure 5.

First, we synchronized the two sensors and the video labels with the help of the aforementioned heel drops to get annotated acceleration signals. Second, we used a 20 Hz low-pass fourth-order Butterworth filter on our dataset since human body movements are below 20 Hz [81]. Third, we segmented the time series into non-overlapping one-second windows (50 samples at 50 Hz). Such a windowing technique is often used in machine-learning-based HAR [16,82]. It enables the extraction of several time- and frequency-domain features. Furthermore, it is better suited for CNNs as they work on windows rather than single data points. The majority of annotated labels in a single window are used as the corresponding ground truth. It is important to mention that this strategy can also introduce errors since activities shorter than half of the window size are not considered. The influence of different window sizes on the prediction performance is not easy to determine. To exemplify this, let a test set consist of a 100-s recording (5000 samples at 50 Hz). Windowing with five seconds results in 20 test samples. On the other hand, one-second windows lead to 100 test samples and, therefore, a higher probability of wrong predictions. One possible solution to this problem is to extract features out of windows of different sizes at once. Herrera-Alcántara et al. [38] investigated a promising approach using wavelets of different scales for feature extraction. We think that this is an interesting topic, but it would go beyond the scope of this paper. Banos et al. [83] showed that larger windows are beneficial for complex activities, but not so much for the simple ones we use. Additionally, they report that with a rich feature set (more than two per axis), shorter windows (one to two seconds) exhibit better results. Due to the findings of Banos et al. [83] and to reduce the loss of short activities while providing large enough windows for feature creation, we choose one-second windows for this work. Furthermore, it leads to a five times larger dataset compared to five-second windows, which can be beneficial for deep learning models. These windows are directly used to train the deep learning models as they can learn features from raw data [84]. We stack the windows of the six axes (three for each sensor) above each other, resulting in a 6×50 matrix, used as the input for the deep learning models.

Fourth, for the traditional machine learning models, we extracted time- and frequency-domain features out of each window. We consider eight signals for feature computation, the six axes (three for each sensor), and each sensor’s vector magnitude $\sqrt{x^{2}+y^{2}+z^{2}}$. Inspired by Stewart et al. [12], features of the human’s orientation and movement were separated by computing the gravity and movement component of the raw accelerometer signal. We applied a fourth-order 1 Hz low-pass Butterworth filter to estimate the former component. Subtracting the resulting gravity component from the raw signal provides the movement component. We computed the *mean*, the *median*, the *standard deviation*, the *coefficient of variation*, the *25th*, and *75th percentile*, as well as the *minimum* and *maximum* for each frame of the gravity components, to get orientation information. For the movement components, we computed the *skew*, *kurtosis*, and *signal energy*, as well as the frequency-domain features *frequency-domain magnitudes’ mean*, *frequency-domain magnitudes’ standard deviation*, *dominant frequency*, *dominant frequency’s magnitude*, *spectral centroid*, and *total signal power*. Narayanan et al. [13] showed that cross-sensory features have a strong influence on the final machine learning performance. Hence, we further computed the *axis correlation* between all six axes and between the two vector magnitude signals. In addition, we computed the mean across the two sensors’ gravity components. In total, we generated 161 features for each window. As a fifth and last preprocessing step, we scaled the features through min–max scaling to the range 0–1 in order to avoid large range differences between features. The target of each machine learning model is to learn the twelve labels of our dataset.

## 4. Experiments and Results

The experiments are examined in two stages. First, we performed hyperparameter optimization combined with cross-validation to find reasonable hyperparameters for each machine learning model. Afterward, a leave-one-subject-out cross-validation was carried out to compare the performance metrics between the different machine learning models.

### 4.1. Hyperparameter Optimization

The hyperparameter optimization with cross-validation was carried out by using two randomly chosen subjects of each of the mentioned dataset’s two sessions for testing. The remaining 18 subjects were used for training. By consistently using test subjects of both sessions, we avoid a possible bias towards the larger one. This cross-validation technique results in three iterations, each having different subjects in the test set. We trained each hyperparameter assignment on these three iterations and averaged the results for comparison. We focus here on the average F1-score (across all twelve labels) as a performance metric since it is more robust to class imbalance than the accuracy [83].

We utilized 1D convolutional kernels in the first layers of the two CNN models to enable a single kernel to learn to extract useful information of each axis. The bidirectional LSTM, on the other hand, uses a fully connected input layer for the whole 6×50 window. For the CNN, we tune the learning rate (best: 0.001), the number of kernels, which is the same for each layer (best: 128), the kernel shape/size in each layer (best: [6, 12, 12, 32]), and the number of layers (best: 4). For the multi-resolution CNN, we also tune the learning rate (best: 0.001), the number of kernels in each layer (best: 64), and the number of layers (best: 2). Furthermore, different kernel sizes in the multi-resolution modules are utilized (best: [3, 5, 7, 9]). The learning rate (best: 0.001) and the number of layers (best: 2) are also tuned for the BiLSTM algorithm. Additionally, the number of units in each layer (same for forward and backward) is examined (best: [32, 32]). The number of epochs for each deep learning model is fixed to 80. A dropout layer, with a rate of 0.4 and 0.2 for the CNNs and LSTM, respectively, is used after each layer to mitigate overfitting. The last two hidden layers of each deep learning model are fully connected 512-dimensional layers with ReLU activation. They are followed by the 12-dimensional prediction/output layer with softmax activation. The utilized optimizer is the stochastic gradient descent algorithm, and the categorical cross-entropy is used as the loss function. The validation set of each deep learning model is the same as the test set. Hence, no early stopping is examined. The validation set is only used to monitor the models’ performance after each epoch. After training, we use this information to ensure that no overfitting occurs.

For the k-NN, different numbers of neighbors [1,2,…,12,20,30] are utilized, with the best value of k=11. We used the radial basis function as the kernel function for the SVM. We investigated the regularization parameter *C*, with larger values causing a more substantial penalty on wrongly classified samples (best: 10). Furthermore, we utilized different γ values, a parameter of the radial basis function (best: $\frac{1}{N\cdot \sigma_{X}^{2}}$), with the variance $\sigma_{X}^{2}$ of the training set *X* and the number of features N=161. Bootstrapping is used for the RF classifier. At each node in a decision tree, $\sqrt{N}$ features are randomly sampled to find an optimal split. Gini impurity is used to measure the quality of a split. Different numbers of decision trees are considered for hyperparameter optimization (best: 80). Additionally, different minimum samples required to split a node are examined (best: 10). The learning rate (best: 0.1), the number of decision trees (best: 1024), and the maximal decision tree depth (best: 3) are tuned for the XGB model. The fixed parameters are the regularization parameters λ=1 (L2) and α=0 (L1). Neither bootstrapping nor feature subsampling is performed. The loss function is the multi-class classification error rate.

### 4.2. Leave-One-Subject-Out Cross-Validation

For each of the seven machine learning approaches, we choose the hyperparameters with the highest F1-score to perform a leave-one-subject-out cross-validation (LOSO). Hence, we train each model on 21 subjects of our dataset and test them on the remaining subject. We repeat this 22 times with a different test subject each time. LOSO shows less subject-based bias than other cross-validation methods [12], which is essential as the same activity can differ greatly between subjects [85]. For each iteration, we compute the corresponding confusion matrix. We sum up the resulting 22 matrices to get a single confusion matrix representing all activities in the dataset. This summed confusion matrix is then used to compute the recall, precision, and F1-score. These three metrics averaged across all twelve labels are shown in Table 2. The best results are shown as gray cells. We observe that the SVM shows the best F1-score and recall. It further has the second-best precision. Hence, it can be considered as the best model of our experiments. The second-best model, under consideration of the F1-score, is the XGB, followed by the k-NN. All deep learning approaches have comparably low values in all metrics. The worst model is the RF. The standard deviation is high, independent of the model or the metric.

We are mainly interested in physical activity classification. Some of our labels involve a similar physical activity even though they have a different label. Therefore, we can merge certain labels. In particular, shuffling, transport (standing), and standing are fused to the same physical activity standing. Sitting and transport (sitting) are merged into sitting. This merging is achieved by summing up the corresponding columns and rows in the summed confusion matrix, respectively, resulting in nine activity labels. Table 3 gives a further overview of each model’s average F1-score, precision, and recall, focusing on the nine physical activities. The performance of all models increased considerably for each metric. Furthermore, a lower standard deviation is observable. Again, the best model is the SVM. However, the deep learning models benefit from the label merging as they exhibit the highest performance increase, e.g., the multi-resolution CNN now has the second-highest F1-score.

**Figure 6:**
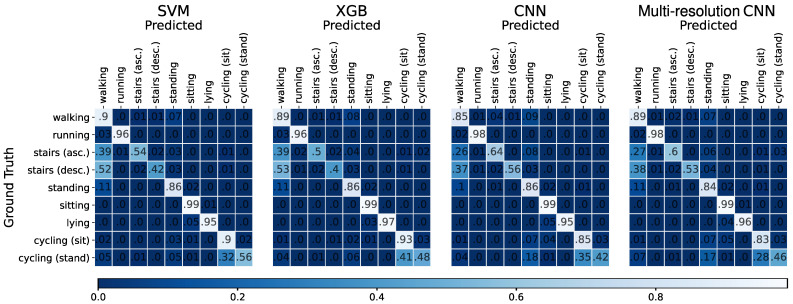
This figure shows four summed confusion matrices of the leave-one-subject-out cross-validation. The four considered models are the two best traditional machine learning approaches, SVM (left) and XGB (right of SVM), as well as the two best deep learning models CNN (left of multi-resolution CNN) and multi-resolution CNN (left). The rows show the ground truth labels and the columns the predictions. Additionally, the matrices are normalized such that each row sums up to one. The diagonal represents the proportion of correctly classified samples. The leading zero of each entry is removed.

Figure 6 shows the summed confusion matrices of the two best traditional machine learning models (SVM and XGB) and the two best deep learning models (CNN and multi-resolution CNN). The rows represent the ground truth, and the columns represent the model predictions. The matrices are normalized such that the values of each row sum up to approximately one (with some rounding errors). The diagonal represents the proportion of correctly classified samples. Nearly all activities are well predicted, with the highest value of 99% correctly classified samples for sitting. Lying and running show similar high entries of at least 95%, followed by walking (85–90% correctly predicted samples), cycling (sitting) (83–93%), and standing (84–86%). However, three activities stand out due to their poor results in each model. These are stairs (ascending) (50–64%), stairs (descending) (40–56%), and cycling (standing) (42–56%). The former two are often confused with walking and the latter one with cycling (sitting). The deep learning models distinguish better between stairs (ascending)/stairs (descending) and walking. However, the prediction performance is still low.

## 5. Discussion

Our results show that the SVM is the best model. However, all trained methods have similar high performance, indicating well-chosen hyperparameter assignments. Additionally, all seven models seem to struggle with the same issues. First, when the activities are not merged (see Table 2), a high standard deviation in all metrics is observable. As the results are averaged across the twelve labels, this high standard deviation indicates a big difference in the prediction performance of different activities. Hence, some labels can be well predicted, others not. We assume that the similar nature between certain activities causes this. Merging the classes results in lower standard deviations, which confirms our assumption. Second, in general, stairs (ascending), stairs (descending), and cycling (standing) are often misclassified, independent of the model. This independence indicates that the confusions are rather an aspect of the dataset and not the machine learning models. The fact that both the deep learning and traditional machine learning models have this issue strengthens this assumption, as both use different signal representations. We assume that the main reason for the low performance is that these three labels exhibit the lowest number of minutes in the dataset. Hence, future work can tackle this issue by developing machine learning models that can handle class imbalances, e.g., by performing class weighting to strengthen the influence of minor classes. The deep learning models seem to distinguish stair walking and walking better than the XGB and SVM. This aspect indicates that certain features necessary to differentiate these activities are not captured by our 161 features, requiring the investigation of more features in future work. However, the deep learning models do not show the best results. This might be caused by the relatively small dataset, compared to datasets of other fields such as computer vision or automatic speech recognition, where deep learning approaches excel. However, recording similar large HAR datasets is not trivial. Hence, for future deep learning-based HAR, we recommend using models that do not require a vast dataset.

Our dataset shows a strong resemblance to the HUNT4 data regarding used sensors, sensor positions, and recordings in free living. With its professionally annotated activities, it serves as a qualified training dataset to train HAR machine learning models that can be used for physical activity-based public health studies using the HUNT4 data.

## 6. Conclusions

An accelerometer-based HAR dataset needs two essential properties for physical activity behavior-based public health research. First, accurate acceleration measurements are required, including fixed sensor positions, noise robustness, and professionally annotated physical activities. Second, the data need to be recorded under free-living conditions. To the best of our knowledge, there is currently no benchmark accelerometer-based HAR dataset publicly available that has both properties. We make two contributions in this work. First, we fill this gap in existing benchmarks by introducing the human activity recognition Trondheim dataset (HARTH), a professionally annotated dataset, recorded under free-living conditions using two accelerometers attached to the participants’ back and thigh. Our second contribution is the training of seven baseline machine learning models. The HARTH dataset and the source code of our models are publicly available. Thus, they can be used as a reference for further development in future research.

The window size plays a crucial role in the HAR performance. Future work can investigate the usage of dynamic windows as well as the temporal relation between windows. Another approach is to extract features of different window sizes at once, similar to the work of Herrera-Alcántara et al. [38]. A challenging aspect of HARTH is that the classes are highly imbalanced, which is not tackled in this work. In future research, techniques such as class balancing or augmentation can improve the results.

Due to the high-quality recordings and annotations of our dataset, as well as its challenging nature, we provide a promising basis for different research directions such as data augmentation, class balancing, and single data sample prediction. Our results show that there is still room for improvement for researchers to develop innovative machine learning approaches to facilitate a more precise human activity recognition in free-living environments.

## Figures and Tables

**Table 1:** This table shows the main characteristics of eight different publicly available HAR accelerometer-based datasets, and our HARTH. We consider the symbol “#” as an abbreviation for “number of”, “PAs” for “physical activities” and “accelero.” for “accelerometers”.

Name	#Labels	#PAs	#Subjects	#Accelero.	Sensor Type	Annotation
Real-life-HAR [24]	4	2	19	1	Smartphone	User
SHL [36,37]	8	5	3	4	Smartphone	User and expert
HASC-PAC2016 [25]	6	6	81	1	Smartphone	User
WISDMv2.0 [26,27]	6	6	225	1	Smartphone	User
DailyLog [28]	19	7	7	2	Smartphone & Smartwatch	User
ExtraSensory [29]	51	8	60	2	Smartphone & Smartwatch	User
TMD [30]	5	3	13	1	Smartphone	User
SDL [38]	10	4	8	1	Smartwatch	User
HARTH (ours)	12	9	22	2	Axivity AX3	Human experts

**Table 2:** This table shows the recall, precision, and F1-score of the leave-one-subject-out cross-validation, averaged across all twelve labels, with the corresponding standard deviations. The best results are shown as gray cells. The term “mCNN” is an abbreviation for “multi-resolution CNN”.

	k-NN	SVM	RF	XGB	BiLSTM	CNN	mCNN
Recall	0.60±0.36	0.63±0.34	0.59±0.39	0.62±0.36	0.61±0.37	0.61±0.38	0.61±0.37
Precision	0.70±0.28	0.70±0.29	0.66±0.33	0.69±0.31	0.64±0.35	0.69±0.30	0.65±0.33
F1-score	0.63±0.33	0.66±0.32	0.61±0.36	0.64±0.35	0.62±0.36	0.61±0.36	0.62±0.36

**Table 3:** This table shows the average recall, precision, and F1-score of the leave-one-subject-out cross-validation. Twelve labels are merged into nine physical activities by summing up the corresponding rows/columns of the summed confusion matrix. The best results are shown as gray cells. The term “mCNN” is an abbreviation for “multi-resolution CNN”.

	k-NN	SVM	RF	XGB	BiLSTM	CNN	mCNN
Recall	0.75±0.26	0.79±0.22	0.73±0.31	0.78±0.24	0.77±0.21	0.79±0.20	0.79±0.20
Precision	0.83±0.15	0.85±0.13	0.83±0.14	0.84±0.15	0.81±0.17	0.82±0.17	0.82±0.15
F1-score	0.78±0.22	0.81±0.18	0.76±0.25	0.80±0.20	0.79±0.19	0.80±0.19	0.80±0.18

## Data Availability

The dataset presented in this article is publicly available on https://github.com/ntnu-ai-lab/harth-ml-experiments (accessed on 16 November 2021).

## Appendix A

**Table A1:** The definitions of all twelve activities used during annotation.

Activity	Definition
Sitting	When the person’s buttocks is on the seat of the chair, bed, or floor. Sitting can include some movement in the upper body and legs; this should not be tagged as a separate transition. Adjustment of sitting position is allowed.
Standing	Upright, feet supporting the person’s body weight, with no feet movement, otherwise this could be shuffling/walking. Movement of upper body and arms is allowed. If feet position is equal before and after upper body movement, standing can be inferred. Without being able to see the feet, if upper body and surroundings indicate no feet movement, standing can be inferred.
Lying	The person lies either on the stomach, on the back, or on the right/left shoulder. Movement of arms, feet, and head is allowed.
Walking	Locomotion towards a destination with one stride or more, (one step with both feet, where one foot is placed at the other side of the other). Walking could occur in all directions. Walking along a curved line is allowed.
Running	Locomotion towards a destination, with at least two steps where both feet leave the ground during each stride. Running can be inferred when trunk moves forward is in a constant upward-downward motion with at least two steps. Running along a curved line is allowed.
Stairs (asc./desc.)	Start: Heel-off of the foot that will land on the first step of the stairs. End: When the heel-strike of the last foot is placed on flat ground. If both feet rests at the same step with no feet movement, standing should be inferred.
Shuffling	Stepping in place by non-cyclical and non-directional movement of the feet. Includes turning on the spot with feet movement not as part of walking bout. Without being able to see the feet, if movement of the upper body and surroundings indicate non-directional feet movement, shuffling can be inferred.
Cycling (sitting)	Pedaling while the buttocks is placed at the seat. Cycling starts at first pedaling, or when the bike is moving while one/both feet are on the pedal(s). Cycling ends when the first foot is in contact with the ground. If one/both feet are placed on the pedal(s), the buttocks is placed at the seat, with no pedaling and the bike is standing still, this should be tagged as sitting.
Cycling (standing)	Standing with both feet on the pedals, while riding a bike. Cycling (standing) starts when the buttocks leave the seat, and ends when the buttocks is placed on the seat.
Transport (sitting)	When sitting in a bus/car/train among others.
Transport (standing)	When standing in a bus/train among others. Movement of feet while standing is allowed and should not be tagged separately.
